# Supplementary material for: Knowledge about mother to child transmission of HIV/AIDS, its prevention and associated factors among reproductive-age women in sub-Saharan Africa: Evidence from 33 countries recent Demographic and Health Surveys
Source: PLoS One. 2021 Jun 11;16(6):e0253164. doi: 10.1371/journal.pone.0253164 (PMC8195361; doi:10.1371/journal.pone.0253164)
Supplement: S1 Table — (DOCX) [file pone.0253164.s001.docx]

**S1 Table. Percentage distribution of study participants by country**

| Country | Year | Percentage of study participants (%) |
| --- | --- | --- |
| Angola | 2015/16 | 2.9 |
| Burkina Faso | 2010 | 4.21 |
| Benin | 2017/18 | 1.72 |
| Burundi | 2016/17 | 4.6 |
| DR Congo | 2013/14 | 4.08 |
| Congo | 2011/12 | 2.6 |
| Cote D’Ivoire | 2012 | 2.29 |
| Cameroon | 2018/19 | 3.41 |
| Ethiopia | 2016 | 3.61 |
| Gabon | 2012 | 2.25 |
| Ghana | 2014 | 2.2 |
| Gambia | 2013 | 2.52 |
| Guinea | 2018 | 1.84 |
| Kenya | 2014 | 3.94 |
| Comoros | 2012 | 1.05 |
| Liberia | 2013 | 2.15 |
| Lesotho | 2014 | 1.77 |
| Madagascar | 2008/09 | 1.92 |
| Mali | 2018 | 1.95 |
| Malawi | 2011 | 6.34 |
| Mozambique | 2011 | 3.27 |
| Nigeria | 2018 | 9.91 |
| Niger | 2012 | 2.01 |
| Namibia | 2013 | 2.45 |
| Rwanda | 2014/15 | 3.79 |
| Sierra Leone | 2013 | 3.7 |
| Sao Tome Principe | 2008/09 | 0.63 |
| Chad | 2014/15 | 1.04 |
| Togo | 2013/14 | 2.27 |
| Uganda | 2016 | 5.11 |
| South Africa | 2016 | 2.12 |
| Zambia | 2018/19 | 3.62 |
| Zimbabwe | 2015 | 2.74 |
