# Supplementary material for: Knowledge about mother to child transmission of HIV/AIDS, its prevention and associated factors among reproductive-age women in sub-Saharan Africa: Evidence from 33 countries recent Demographic and Health Surveys
Source: PLoS One. 2021 Jun 11;16(6):e0253164. doi: 10.1371/journal.pone.0253164 (PMC8195361; doi:10.1371/journal.pone.0253164)
Supplement: S2 Table — (DOCX) [file pone.0253164.s002.docx]

**S2 Table. Prevalence of Knowledge about mother to child transmission of HIV/AIDS and its prevention among reproductive age women in Sub-Saharan Africa.**

| **Country** | **Year of the survey** | **Prevalence of Knowledge about mother to child transmission of HIV/AIDS and its prevention (%)** |
| --- | --- | --- |
| Angola | 2015/16 | 68.64 |
| Burkina Faso | 2010 | 54.86 |
| Benin | 2017/18 | 58.39 |
| Burundi | 2016/17 | 71.08 |
| DR Congo | 2013/14 | 20.82 |
| Congo | 2011/12 | 41.15 |
| Cote D’Ivoire | 2012 | 50.66 |
| Cameroon | 2018/19 | 64.6 |
| Ethiopia | 2016 | 46.83 |
| Gabon | 2012 | 62.83 |
| Ghana | 2014 | 56.61 |
| Gambia | 2013 | 56.36 |
| Guinea | 2018 | 45.73 |
| Kenya | 2014 | 41.36 |
| Comoros | 2012 | 13.56 |
| Liberia | 2013 | 54.57 |
| Lesotho | 2014 | 65.19 |
| Madagascar | 2008/09 | 31.15 |
| Mali | 2018 | 46.09 |
| Malawi | 2011 | 71.33 |
| Mozambique | 2011 | 67.26 |
| Nigeria | 2018 | 59.25 |
| Niger | 2012 | 41.75 |
| Namibia | 2013 | 66.57 |
| Rwanda | 2014/15 | 62.47 |
| Sierra Leone | 2013 | 57.07 |
| Sao Tome Principe | 2008/09 | 24.8 |
| Chad | 2014/15 | 37.9 |
| Togo | 2013/14 | 64.07 |
| Uganda | 2016 | 56.76 |
| South Africa | 2016 | 76.02 |
| Zambia | 2018/19 | 59.71 |
| Zimbabwe | 2015 | 70.54 |
| Over all prevalence | 56.21% (95% CI: 56.05-56.38) | |
