# Supplementary material for: Knowledge about mother to child transmission of HIV/AIDS, its prevention and associated factors among reproductive-age women in sub-Saharan Africa: Evidence from 33 countries recent Demographic and Health Surveys
Source: PLoS One. 2021 Jun 11;16(6):e0253164. doi: 10.1371/journal.pone.0253164 (PMC8195361; doi:10.1371/journal.pone.0253164)
Supplement: Attachment — Submitted filename: Rebuttal letter.docx [file pone.0253164.s004.docx]

**Date: May 28, 2021**

**Rebuttal letter**

**Title:** Knowledge about mother to child transmission of HIV/AIDS, its prevention and associated factors among reproductive-age women in sub-Saharan Africa: Evidence from 33 countries recent Demographic and Health Surveys

**Manuscript number:** PONE-D-20-37111R1

Dear editors and reviewers, thank you for your constructive comments and suggestions. Here, below, is the authors’ point-by-point response for the comments raised.

**Response to questions regarding Journal Requirements**

Please review your reference list to ensure that it is complete and correct. If you have cited papers that have been retracted, please include the rationale for doing so in the manuscript text, or remove these references and replace them with relevant current references. Any changes to the reference list should be mentioned in the rebuttal letter that accompanies your revised manuscript. If you need to cite a retracted article, indicate the article’s retracted status in the References list and also include a citation and full reference for the retraction notice.

Authors’ Response: Thank you for the comment. The author have confirmed that there is no retracted article in the reference list and we have checked and rewritten reference 1 and 2 accordingly (see the revised manuscript).

**Response for Additional Editor Comments**

Line 57, for some readers, particularly in non-generalized epidemics context, the option B+ may be difficult to understand; please provide an explanation for the term.

Authors’ Response: **Option B+** is offering immediate antiretroviral therapy for mothers living with HIV to prevent the vertical transmission HIV/AIDS regardless of their CD4 count. That is, option B+ is initiation of antiretroviral therapy for all pregnant mothers, regardless of their CD4 count, to PMTCT of HIV/AIDS. In the revised manuscript, we have consider such points.

Line 72-74 is only a one-sentence paragraph; please integrate the line into the next paragraph.

Authors’ Response: Thank you. We have considered your comment (we have added the lines into the next paragraph) in the revised manuscript.
